# Changes in the pharmacological management of rheumatoid arthritis over two decades

**DOI:** 10.1093/rheumatology/keaa892

**Published:** 2021-01-06

**Authors:** Samantha S R Crossfield, Maya H Buch, Paul Baxter, Sarah R Kingsbury, Mar Pujades-Rodriguez, Philip G Conaghan

**Affiliations:** 1 Leeds Institute of Rheumatic and Musculoskeletal Medicine; 2 Leeds Institute for Data Analytics, University of Leeds, Leeds; 3 Centre for Musculoskeletal Research, School of Biological Sciences, University of Manchester, Manchester; 4 Leeds Institute of Cardiovascular and Metabolic Medicine, University of Leeds; 5 NIHR Leeds Biomedical Research Centre; 6 Leeds Institute of Health Sciences, University of Leeds, Leeds, UK

**Keywords:** rheumatoid arthritis, electronic health records, disease-modifying anti-rheumatic drugs, corticosteroids, trends

## Abstract

**Objectives:**

To assess whether modern management of RA has reduced the prescription of oral corticosteroids and NSAIDs and to evaluate use of pharmacological prophylaxis strategies.

**Methods:**

Using the Clinical Practice Research Datalink, we explored long-term (≥3/12 months; ≥6/12 in sub-analyses) DMARD, corticosteroid and NSAID prescribing (annually, in the year post-diagnosis and across the patient’s life course to 15 years post-diagnosis), annual proportion with co-prescribing for prophylaxis of associated bone (corticosteroids, women only) and gastrointestinal (NSAIDs) comorbidity.

**Results:**

Reported incidence of RA was 5.98 (0.37) per 10 000 person-years and prevalence was 0.91% (0.014) in 2017. In 71 411 RA patients, long-term DMARD prescribing initially rose post-diagnosis from 41.6% in 1998 to 67.9% in 2009. Corticosteroid prescribing changed little, overall [22.2% in 1998, 19.1% in 2016; incident risk ratio (IRR) 0.92, 95% CI: 0.82, 1.03] and across the life course from the first to fifteenth year (22.2% to 16.9%). NSAID prescribing declined from 57.7% in 1998, and significantly so from 2008, to 27.1% in 2016 (IRR 0.50, 95% CI: 0.44, 0.56). This continued across the life course (41.2% to 28.4%). Bone prophylaxis increased to 68.1% in 2008 before declining to 56.4% in 2017; gastrointestinal prophylaxis increased from 11.5% in 1998 to 62.6% in 2017. Sub-analyses showed consistent patterns.

**Conclusion:**

Despite modern treatment strategies, corticosteroid prescribing in RA patients remains substantial and persists beyond 6 months once initiated. Rheumatologists need to determine causes and develop strategies to reduce corticosteroid use to minimize adverse event occurrence.

**Rheumatology key messages**

- Despite modern RA treatment strategies, long-term prescribing of corticosteroids remains substantial.
- The proportion of RA patients receiving corticosteroids persists across the life course, with suboptimal bone prophylactic therapy.
- Long-term corticosteroid use has implications for RA comorbidities and infection susceptibility (including COVID-19).

## Introduction

Modern treatment strategies for RA employ early initiation of disease-modifying anti-rheumatic drugs (DMARDs) and short-term concomitant corticosteroids to suppress inflammation (especially in early RA), with NSAIDs offering symptomatic relief [1]. These strategies control inflammation through a treat-to-target approach, which is associated with improved patient outcomes [2]. Rheumatologists initiate prescribing and then co-manage patients with general practitioners (GPs).

Short-term corticosteroid therapy (e.g. 2–3 months in UK guidelines, 6 months in COBRA-type regimens) is recommended in early RA, when initiating or changing DMARDs, with tapered withdrawal of corticosteroids guided by response and risk factors [1, 3, 4]. Long-term DMARD therapy should limit inflammation and the symptoms that underpin continued corticosteroid and NSAID prescribing. The latter agents may mask uncontrolled disease activity and are associated with substantial long-term risks (including cardiovascular, bone and gastrointestinal disorders) even at low doses, particularly among the elderly population with comorbidities [5–12]. Guidelines recommend prophylaxis co-prescribing: proton-pump inhibitors (PPIs) to mitigate against gastrointestinal adverse effects of NSAIDs; and bone-protective treatment when prescribing ≥7.5 mg of prednisolone daily for ≥3 months, to prevent osteoporotic fractures [13–17].

We investigated trends in the pharmacological management of RA over 20 years to determine whether modern use of DMARDs and tight control of inflammation has resulted in less long-term use of corticosteroids and NSAIDs. We also aimed to assess patterns in prophylactic therapy co-prescribing.

## Methods

We report on a retrospective observational study, following the Strengthening the Reporting of Observational Studies in Epidemiology guidelines (Supplementary Table S1, available at *Rheumatology* online) [18]. The Clinical Practice Research Datalink (CPRD) Independent Scientific Advisory Committee approved the protocol (18_082). There was no patient-public involvement in the study; dissemination of results to study participants is not possible.

### Data source

We used the April 2018 update of the CPRD GOLD dataset, containing 17.6 million electronic health records (EHRs) from 734 UK GP practices. Data undergoes quality assessment and patients have a comparable age, sex and ethnicity profile to the national census statistics and a body mass index distribution to the NHS Health Survey for England [19–21].

### Study population

The eligible population had ≥1 day of continuous registration during the study period (1 January 1998–1 April 2018). Patients with a juvenile RA diagnosis or diagnosis of RA before 18 years of age, of unknown sex or with records flagged as ‘unacceptable’ quality for research were excluded. Patients contributed data from the latest of: the study start date, becoming aged ≥18 years, and having 1 year of CPRD good quality (‘up to standard’) registration [19]. Follow-up ended at the study end date, last data collection from the GP practice, practice deregistration, death, or becoming aged ≥101 years.

We identified diagnoses via Read Version 2 codes (Supplementary Tables S2 and S3, available at *Rheumatology* online). RA codes in CPRD were previously validated (∼80% positive predictive value) [22, 23]. To improve certainty of RA diagnosis, we also used more specific definitions in two sensitivity analyses; ≥2 RA diagnoses at least 6 months apart; and an RA diagnosis with a subsequent DMARD prescription before April 2018.

To assess whether trends in non-DMARD medication prescribing were related to their diagnosis of RA, for each patient with a diagnosis of RA during the study period, five non-RA patients were randomly selected and matched by sex and date of birth ±5 years from patients registered at the same practice on the index date of the RA diagnosis. Non-RA patients had at least 6 months following the index date with no RA diagnosis; their follow-up ended if RA was subsequently diagnosed.

### Outcomes

The outcomes were the annual RA incidence and prevalence, the annual proportion of RA patients receiving any or long-term DMARD, oral corticosteroid or NSAID prescribing, variation in oral corticosteroid or NSAID prescribing, and the annual proportion of RA patients receiving prophylaxis co-prescribing alongside long-term low or high prednisolone dose and/or NSAID prescribing. Variation in prescribing was compared by RA diagnosis, year, sex, age (18–29 then 10-year bands up to 99), GP practice, and socioeconomic deprivation.

We defined long-term prescribing as ≥90 days (≥180 in sub-analyses) total prescription duration within 12 months. Low and high prednisolone dose is defined in guidelines as <7.5 mg and ≥7.5 mg, respectively [16]. Socioeconomic deprivation was defined using Index of Multiple Deprivation quintiles.

### Statistical analyses

Baseline cohort characteristics were described for the prevalent RA, matched RA and non-RA cohorts. Outcome measures were stratified by sex, age and geographical area where there was patient representation from ≥5 GP practices per area [19]. We reported annual trends in patient outcomes between 1 January 1997 and 31 December 2017 and calculated age as on 1 July. Sensitivity analyses ran until 2016 to enable >16 months of follow-up for the additional coding and prescribing to occur.

We calculated crude annual and period incidence rates per 10 000 person-years with 95% CIs for patients ‘at-risk’, i.e. having no RA diagnosis and ≥1 year of prior GP registration at the start of that time period [24, 25]. We divided the incident RA patient count by the total person-years of follow-up. We calculated crude point (1 July of each calendar year) and period prevalence percentages with 95% CI. We calculated the annual percentage changes (APCs) and performed sensitivity analyses.

We identified medication prescriptions using British National Formulary terms (Supplementary Table S4, available at *Rheumatology* online). We calculated annual mean counts of DMARD, oral corticosteroid and NSAID prescriptions per person-year with APC, standardized for follow-up duration, and performed sensitivity analyses. We calculated prescription durations (Supplementary Data S1, available at *Rheumatology* online) and the annual and period percentage of RA and non-RA patients with long-term prescribing, amongst patients with ≥90 days follow-up in that time frame (year or study period). Similarly, for incident patients in each year, we determined the annual and period percentage with long-term prescribing [individually and in combination (e.g. DMARD and corticosteroid)] in the first year post-diagnosis (1998–2016). For incident patients we calculated the percentage with long-term prescribing in each year post-diagnosis up to the fifteenth year (among patients having ≥90 days follow-up in each year). In sub-analyses, we investigated prescribing as above for patients with ≥1 and ≥180 days of prescribing, amongst patients with ≥1 and ≥180 days follow-up in that time frame (year or study period).

We used Poisson regression with (log) person time as the offset and GP practice as a random intercept, to analyse changes in prescribing by calendar year, sex and age while controlling for the other variables (Supplementary Data S2, available at *Rheumatology* online). A sub-analysis included socioeconomic status where this was recorded. We determined the final coefficient inclusion using the Akaike information criteria, Hausman test and comparison of the coefficients and residual deviance. We used quasi-Poisson regression where the dispersion parameter was >1 and comparison with a zero-inflation model where GP practice was included as a random intercept.

For patients with ≥90 days of NSAID medication prescribing in a given year or year post-diagnosis, we calculated the percentage with ≥90 days of PPI prescribed in that year. We assessed the annual proportion with ≥90 days of bone protectant medication prescribing among women with ≥90 days of low or high prednisolone dose prescribed in that year and having no prior osteoporosis diagnosis. We assessed the bone protective agents (bisphosphonates, calcium and vitamin D) separately and in combination. In period calculations, the proportions with ≥90 days of NSAIDs and PPI or prednisolone and bone protectant in any same year were calculated.

R Version 3.6.2, Microsoft SQL 2017 and Microsoft Excel 2016 were used in analyses.

## Results

We identified 71 411 RA patients (44 426 with ≥2 diagnoses; 45 438 with diagnosis and prescribed DMARD) of which 41 198 were matched to 205 990 non-RA patients (Supplementary Fig. S1, available at *Rheumatology* online). The median age at diagnosis was 57 (IQR: 23), 70.0% (49 974) were female and 58.1% (41 509) had socioeconomic deprivation recorded (Supplementary Table S5, available at *Rheumatology* online).

### Incidence and prevalence

**Fig. 1:**
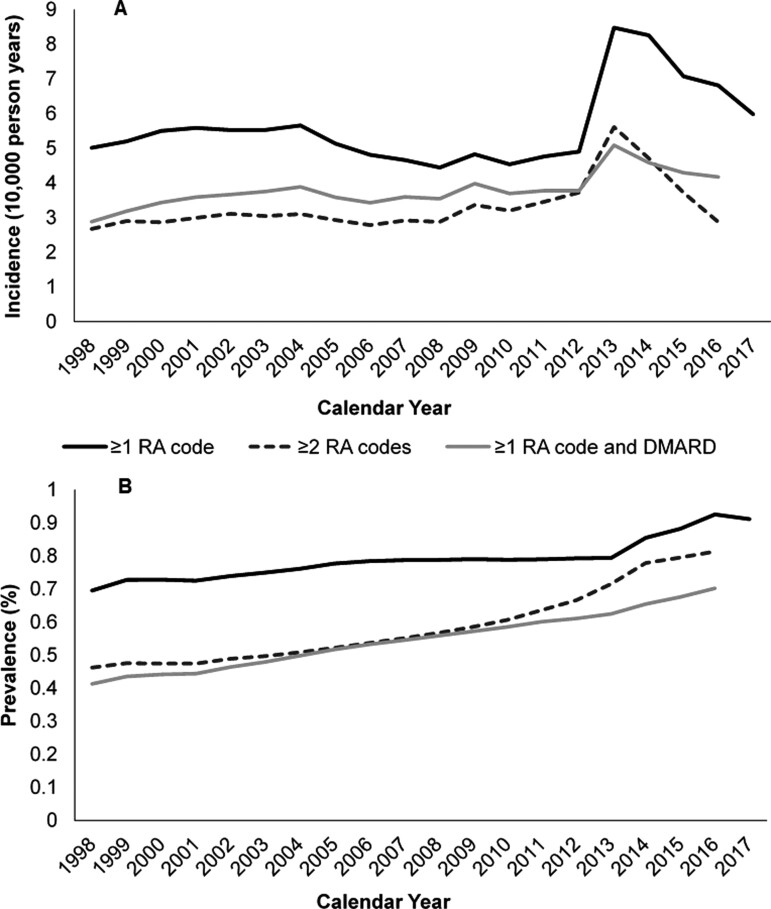
Annual incidence and prevalence (**A**) Annual incidence rate (*N* = 8 022 645); (**B**) annual percentage prevalence (*N* = 7 532 147); in 1998–2017 using three definitions of RA: ≥1 RA diagnostic code; ≥2 RA diagnostic codes at least 6 months apart; ≥1 RA diagnostic code plus a subsequent DMARD prescription.

The period incidence (1998–2017) was 5.57 (0.06) per 10 000 person-years, with 31 768 patients newly diagnosed. The annual incidence was 5.01 (0.36) in 1998 and 5.98 (0.37) in 2017, with a peak at 8.48 (0.32) in 2013 (Fig. 1). The mean APC pre-peak was -0.36; +2.17 and +2.27 in sensitivity analyses. Incidence among women was approximately double that of men, 6.92 (0.60) and 3.01 (0.40) in 1998, respectively; 7.86 (0.58) and 4.33 (0.44) in 2017 (Supplementary Fig. S2, available at *Rheumatology* online). Incidence peaked at age 70–79 [10.53 (1.62) in 1998 and 11.09 (1.52) in 2017] (Supplementary Fig. S3, available at *Rheumatology* online). There was little regional variation excepting for a peak in 2016 (20.03, 3.21) in East England (Supplementary Fig. S4, available at *Rheumatology* online).

The period prevalence was 0.89% (0.01) (Supplementary Table S6, available at *Rheumatology* online); 0.58% (0.01) in sensitivity analyses. Prevalence rose from 0.70% (0.013) in 1998 to 0.91% (0.014) in 2017. The APC rose by mean +1.61 until 2006, before plateauing (mean +0.27) until 2013 (+7.70 in 2013/14), then rising again. Sensitivity analyses demonstrated similar results. Prevalence was highest, and rose steepest, among patients aged 70–99 (2.21%, 0.05) (Supplementary Fig. S5, available at *Rheumatology* online). Differences between women and men remained stable, 6.92 (0.60) and 3.01 (0.40) in 1998, respectively; 7.86 (0.58) and 4.33 (0.44) in 2017; though there was regional variation in prevalence (Supplementary Figs S6 and S7, available at *Rheumatology* online).

### Trends in DMARD prescribing

**Fig. 2:**
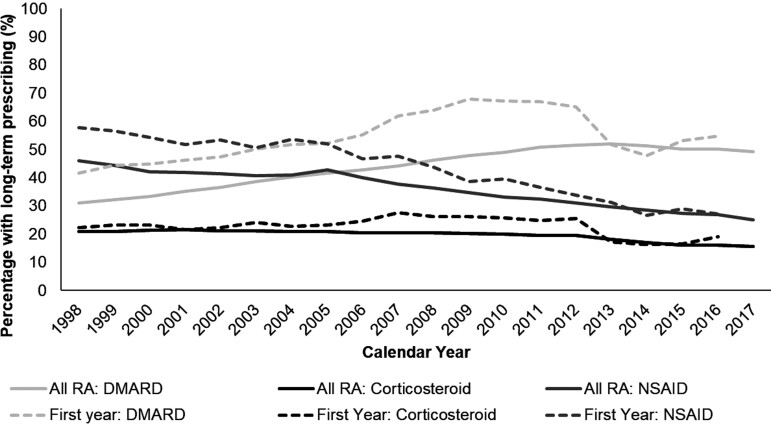
The annual proportion with long-term prescribing The annual percentage (1998–2017) of patients with ≥90 days annual prescribing: all RA patients (*N* = 68 939) and in the first year post-diagnosis (*N* = 29 918).

During follow-up, 59.6% of RA patients had DMARDs prescribed and 55.6% received long-term prescribing (≥90 days in 1 year) for at least 1 year. The mean prescription count per person-year was 3.00 in 1998 and 7.22 in 2017. The proportion with long-term prescribing was 31.0% in 1998, rising on a slowing trajectory to peak at 52.0% in 2013 before falling to 49.3% in 2017 (Fig. 2). Patterns were similar in sub-analyses of ≥1 and ≥180 days prescribing in a given year (Supplementary Figs S8 and S9, available at *Rheumatology* online). Annual proportions were higher among patients with long-term corticosteroid prescribing in a given year (*N* = 22 210): 45.4% in 1998 and 56.1% in 2017.

In the year post-diagnosis, 55.2% had long-term DMARD prescribing. This was 41.6% of patients diagnosed in 1998, rising on a slowing trajectory to peak at 67.9% in 2009 before falling to 54.7% in 2016. Patterns were similar in sub-analyses of ≥1 and ≥180 days prescribing (Supplementary Figs S10 and S11, available at *Rheumatology* online).

### Trends in corticosteroid prescribing

During follow-up, 45.1% of RA patients had prescribed corticosteroids and 32.2% received long-term prescribing for at least 1 year. The mean count of prescriptions per person-year was 2.04 in 1998 and 1.89 in 2017. Among patients prescribed corticosteroids in a given year, the mean prescription count was 8.03 in 1998 and 8.02 in 2017 (Supplementary Fig. S12, available at *Rheumatology* online).

In 1998, 21.0% of RA patients had long-term prescribing, declining (mean APC -1.54) to 15.5% in 2017. Findings from sub-analyses were consistent. The decline was significant between 2013 (IRR 0.87, 95% CI: 0.81, 0.94) and 2017 (IRR 0.75, 95% CI: 0.70, 0.80) (Table 1). Women were slightly less likely to receive long-term corticosteroids than men (IRR 0.96, 95% CI: 0.94, 0.97). Compared with age 18–29 years, prescribing significantly increased with age from 50 (e.g. age 50–59: IRR 1.27, 95% CI: 1.16, 1.39; age 90–99: IRR 1.60, 95% CI: 1.44, 1.78) (Supplementary Fig. S13, available at *Rheumatology* online). Socioeconomic deprivation had no significant effect (data not shown). In the non-RA cohort, 3.8% had long-term corticosteroid prescribing during follow-up, rising from 0.9% in 1998 to 2.0% in 2017 (Supplementary Fig. S14, available at *Rheumatology* online).

**Table 1:** Adjusted incident risk ratios for having long-term medication prescribing. Adjusted incident risk ratios (IRRs) for having ≥90 days annual medication prescribing (all RA patients and in the year post-diagnosis)^†^

	Adjusted IRR (95% CI)			
	Corticosteroid		NSAID	
	All RA patients (*N* = 68 939)	First year post-diagnosis (*N* = 30 799)	All RA patients (*N* = 68 939)	First year post- diagnosis (*N* = 30 799)^†^
**Calendar year**				
1998	1	1	1	1
1999	1.02 (0.95, 1.10)	1.02 (0.89, 1.17)	1 (0.96, 1.05)	0.99 (0.91, 1.08)
2000	1.06 (0.99, 1.13)	1.03 (0.90, 1.17)	0.97 (0.92, 1.01)	0.96 (0.89, 1.05)
2001	1.04 (0.97, 1.12)	0.97 (0.85, 1.10)	0.95 (0.91, 0.99)	0.91 (0.84, 0.99)
2002	1.02 (0.96, 1.08)	1.00 (0.89, 1.12)	0.94 (0.90, 0.97)	0.94 (0.86, 1.01)
2003	1.01 (0.95, 1.07)	1.05 (0.94, 1.18)	0.92 (0.88, 0.95)*	0.89 (0.82, 0.97)
2004	1.00 (0.94, 1.06)	1.00 (0.89, 1.12)	0.92 (0.89, 0.95)*	0.95 (0.88, 1.03)
2005	0.98 (0.92, 1.04)	1.03 (0.92, 1.15)	0.95 (0.92, 0.98)	0.91 (0.85, 0.99)
2006	0.96 (0.91, 1.02)	1.10 (1.00, 1.22)	0.89 (0.86, 0.92)*	0.82 (0.76, 0.89)
2007	0.96 (0.91, 1.02)	1.24 (1.09, 1.41)	0.84 (0.81, 0.88)*	0.83 (0.76, 0.90)
2008	0.96 (0.90, 1.01)	1.18 (1.07, 1.31)	0.81 (0.78, 0.84)*	0.76 (0.70, 0.82)*
2009	0.94 (0.89, 1.00)	1.19 (1.06, 1.33)	0.77 (0.74, 0.80)*	0.68 (0.62, 0.74)*
2010	0.93 (0.87, 0.99)	1.18 (1.08, 1.30)	0.74 (0.71, 0.77)*	0.70 (0.64, 0.76)*
2011	0.92 (0.87, 0.98)	1.13 (1.01, 1.26)	0.73 (0.70, 0.75)*	0.64 (0.58, 0.70)*
2012	0.91 (0.85, 0.98)	1.16 (1.04, 1.31)	0.70 (0.67, 0.72)*	0.60 (0.54, 0.66)*
2013	0.87 (0.81, 0.94)*	0.77 (0.69, 0.86)	0.68 (0.66, 0.70)*	0.56 (0.51, 0.61)*
2014	0.83 (0.77, 0.89)*	0.75 (0.68, 0.83)	0.66 (0.64, 0.68)*	0.48 (0.44, 0.53)*
2015	0.81 (0.76, 0.86)*	0.78 (0.68, 0.90)	0.65 (0.62, 0.67)*	0.53 (0.47, 0.59)*
2016	0.80 (0.75, 0.85)*	0.92 (0.82, 1.03)	0.63 (0.61, 0.65)*	0.50 (0.44, 0.56)*
2017	0.75 (0.70, 0.80)*		0.57 (0.54, 0.60)*	
**Sex**				
Male	1	1	1	1
Female	0.96 (0.94, 0.97)*	0.88 (0.85, 0.92)*	1 (0.99, 1.01)	1.02 (0.99, 1.05)
**Age group**				
18–29	1	1	1	1
30–39	1.07 (0.97, 1.19)	1.05 (0.84, 1.30)	1.15 (1.10, 1.20)*	1.13 (1.03, 1.25)
40–49	1.05 (0.95, 1.15)	1.03 (0.83, 1.27)	1.32 (1.26, 1.37)*	1.17 (1.07, 1.29)
50–59	1.27 (1.16, 1.39)*	1.33 (1.09, 1.62)	1.34 (1.28, 1.39)*	1.16 (1.06, 1.27)
60–69	1.67 (1.52, 1.84)*	1.73 (1.42, 2.11)*	1.24 (1.20, 1.30)*	1.09 (1.00, 1.20)
70–79	2.08 (1.90, 2.27)*	2.35 (1.93, 2.85)*	0.99 (0.95, 1.04)	0.91 (0.83, 1.00)
80–89	2.18 (1.99, 2.40)*	2.6 (2.12, 3.19)*	0.74 (0.70, 0.77)*	0.72 (0.65, 0.81)*
90–99	1.60 (1.44, 1.78)*	2.13 (1.65, 2.75)*	0.56 (0.52, 0.61)*	0.75 (0.61, 0.91)

Note: adjusted for calendar year, sex and age group as appropriate. † GP practice included as a random intercept. *P < 0.001.

In the year post-diagnosis, 22.5% of RA patients had long-term corticosteroid prescribing. This remained stable over the study period (22.2% in 1998 and 19.1% in 2016) and findings from sub-analyses were consistent. Compared with patients aged 18–29 years, the proportion prescribed corticosteroids in the year post-diagnosis increased with age from 50–99 (e.g. age 50–59: IRR 1.33, 95% CI: 1.09, 1.62; age 90–99: IRR 2.13, 95% CI: 1.65, 2.75). Women were less likely to receive corticosteroids in the year post-diagnosis (IRR 0.88, 95% CI: 0.85, 0.92). Socioeconomic deprivation had no significant effect.

### Trends in NSAID prescribing

During follow-up, 69.0% of RA patients had prescribed NSAIDs and 54.4% received long-term prescribing for at least 1 year. The mean count of prescriptions per person-year fell from 4.17 in 1998 to 1.96 in 2017. The proportion with long-term prescribing was 45.9% in 1998 and declined (mean APC -3.10) to 25.1% in 2017, with sub-analyses showing similar patterns. Compared with age 18–29 years, prescribing increased significantly with age until 50–59 (e.g. 50–59: IRR 1.34, 95% CI: 1.28, 1.39) before decreasing with older age. There was no sex difference but long-term prescribing was greater among the least socioeconomic deprived patients (quintile 5 compared with 1: IRR 1.04, 95% CI: 1.02, 1.06). In the non-RA cohort, 19.7% had long-term NSAID prescribing during follow-up, rising from 6.4% in 1998 to 8.4% in 2017.

In the year post-diagnosis, 42.1% had long-term prescribing. This declined from 57.7% in 1998 to 27.1% in 2016, with sub-analyses showing similar patterns. The decline was significant between 2008 and 2016 (e.g. 2008: IRR 0.76, 95% CI: 0.70, 0.82; 2016: IRR 0.50, 95% CI: 0.44, 0.56). GP practice accounted for slight variability in prescribing in the year post-diagnosis (variance: 0.01, standard deviation: 0.11, Hausman *P* = 0.12). There was no sex or socioeconomic deprivation difference but a trend towards lower prescribing with increasing age.

### Prescribing over the life course

**Fig. 3:**
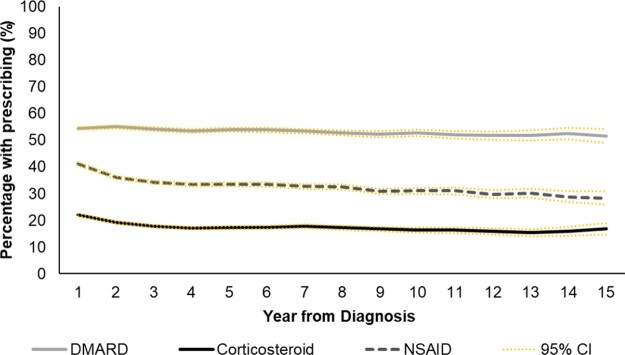
The proportion with long-term prescribing in the 1–15 years post-diagnosis Percentage of incident RA patients with ≥90 days annual prescribing in the 1–15 years post-diagnosis, with 95% CI (*N* = 30 807).

For incident RA patients, 16.5% (*n* = 6604) had 10 years follow-up and 3.0% (*n* = 1460) had 15 years. The proportion with long-term DMARD prescribing did not change significantly over the life course; 54.4% (53.9–55.0%) and 51.6% (48.9–54.3%) in the first and fifteenth year, respectively; although there was a declining trend (mean APC -0.37) (Fig. 3, Supplementary Table S7, available at *Rheumatology* online). For corticosteroids the proportion declined from 22.2% (21.7–22.6%) to 16.9% (14.9–18.9%) and for NSAIDs from 41.2% (40.6–41.7%) to 28.4% (25.9–30.8%). Most of the decline occurred by year 3 [mean APC -10.20 (corticosteroids) and -8.66 (NSAIDs)]. Assessments of combination prescribing showed consistent patterns (Supplementary Fig. S15, available at *Rheumatology* online). Sub-analyses showed similar patterns, excepting a delay in patients receiving ≥180 days of DMARDs until the second year post-diagnosis (Supplementary Figs S16 and S17, available at *Rheumatology* online).

### Prophylaxis co-prescribing

**Fig. 4:**
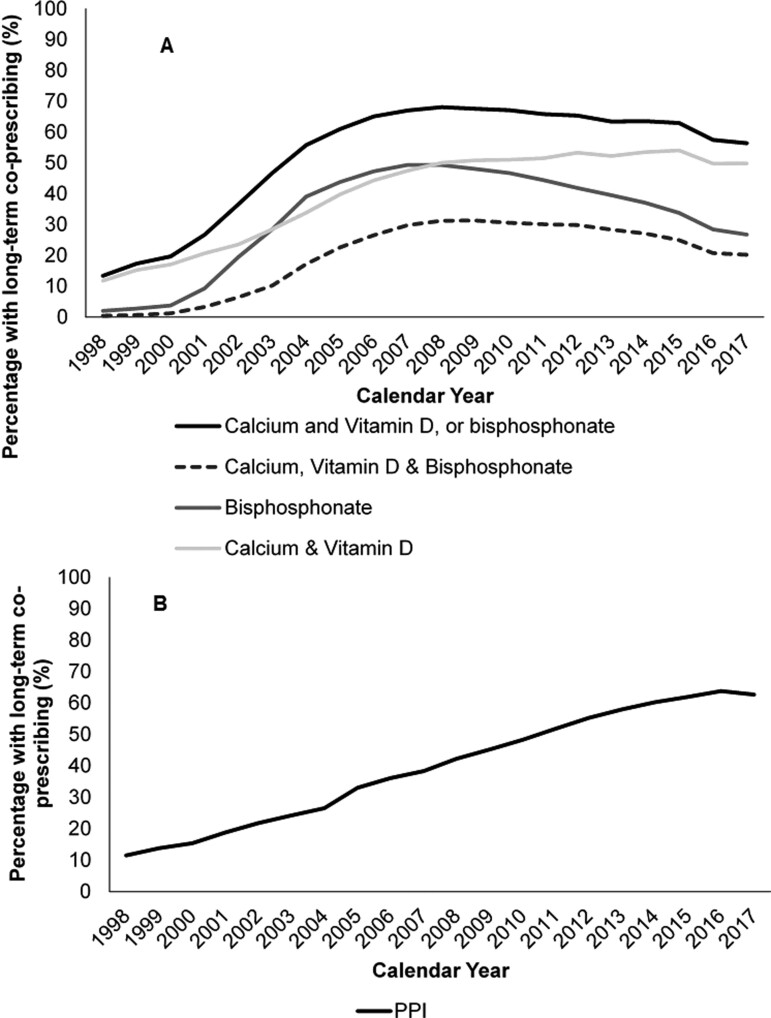
The annual proportion with RA medication and protectant The percentage of RA patients with ≥90 days of annual RA medication and protectant (1998–2017): (**A**) corticosteroid and bone protectant (bisphosphonate, calcium and vitamin D) (*N* = 14 314); (**B**) NSAID and PPI (*N* = 38 480).

During follow-up, 14 314 women with no evidence of osteoporosis prior to RA had long-term prednisolone in a year. Of these, in 1998, 2.1% were prescribed long-term bisphosphonate; 11.8% calcium and vitamin D and 13.4% calcium and vitamin D or bisphosphonate, rising to 26.8%; 49.8% and 56.4% in 2017 (Fig. 4). Long-term bisphosphonate prescribing rose steeply to 49.4% in 2007 before slowly declining, especially among patients aged ≥60 years (Supplementary Fig. S18, available at *Rheumatology* online). The patterns were comparable for high (*n* = 5952) and low (*n* = 13 061) prednisolone dose cohorts (Supplementary Figs. S19 and S20, available at *Rheumatology* online), [e.g. 59.9% (95% CI: 54.9, 65.0) and 55.4% (95% CI: 52.8, 58.1) with calcium and vitamin D or bisphosphonate in 2017, respectively]. Among 38 480 patients with long-term NSAID prescribing, 50.5% had PPI prescribed long-term in the same year. This rose (mean APC +9.52) from 11.5% in 1998 to 62.6% in 2017. The APC declined over time from +19.82 in 1998/9 to -1.75 in 2016/17.

## Discussion

This study demonstrated little change in corticosteroid prescribing in RA patients in the UK by GPs, and no change in the year post-diagnosis despite modern treatment strategies. Although the decline in corticosteroid prescription was significant across the first 3 years post-diagnosis, prescribing remained substantial 3 years post-diagnosis (17.9%) and persisted for the duration of the study, particularly in older age groups. NSAID prescribing halved among RA patients, predominantly driven by changing practice for newly diagnosed patients, though remained substantial (34.3% at 3 years post-diagnosis). Additionally, the increased DMARD prescribing in the year post-diagnosis plateaued from 2009. Improvements in prophylaxis co-prescribing remain suboptimal. In elderly and comorbid populations treated with DMARD immune suppressants, persistent corticosteroid exposure with attendant immune suppression is particularly concerning and pertinent in the context of infectious diseases including COVID-19 and tuberculosis [26].

Our prevalence estimates of RA of ∼0.5–1%, higher among women and increasing with age, are consistent with older studies [25, 27]. Abishek *et al.* using CPRD data, reported lower incidence and prevalence (1990–2014) and declining incidence, which we did not find, especially in sensitivity analyses [25]. This may be due to differences in defining RA. In the UK, GPs receive payment for using specific RA codes to maintain a registry of RA patients and perform annual review and risk assessments [28]. Some of these codes were not used to define RA by Abishek *et al.* although they were in our study. This would make some patients, and particularly those diagnosed in rheumatology clinics and annually reviewed by GPs, less likely to be included in the previous study. Further, we included codes naming RA in specific joints and excluded patients without 1 year of ‘up to standard’ registration.

The proportion of patients with DMARD, corticosteroid and NSAID prescribed during follow-up was comparable to older studies [29–32]. However, we show that corticosteroid prescribing has persisted in the current treat-to-target era, even after the first year of diagnosis. While some therapy strategies continue corticosteroid prescribing beyond the 3- and 6-month definitions of long-term used here [33], we found persistence even 15 years post-diagnosis. As observed in other immune-mediated inflammatory diseases (e.g. polymyalgia rheumatica), corticosteroids may relieve symptoms (e.g. regional musculoskeletal complaints) not necessarily relating to activity of the index disease [34, 35]. This likely perpetuates corticosteroid prescribing despite guidelines and trial-based evidence of associated diabetes, hypertension and cardiovascular disease risk in RA patients [9, 10, 12]. It may also mask symptoms of poor RA disease control. While the difficulties in corticosteroid tapering, including adrenal suppression, are well recognized, there are reported favourable outcomes from discontinuing corticosteroids after 34 weeks in early RA [36]. Intramuscular corticosteroids provide a fixed tapered dose and may be useful for short-term use [4]. Clinicians need to recognise the unmet need for pain control, which can contribute to disease activity scores, and assess whether joint pain is due to RA or other conditions [37–39].

While studies have reported low bone-protectant co-prescribing (<15% between 1991 and 1997) and GI-protectant co-prescribing (10% between 2001 and 2003) in the general population [40, 41], this study shows temporal trends in RA patients. Declining NSAID prescribing among RA patients and increasing GI prophylaxis (especially from 2005) reflects rising awareness of NSAID toxicity through the early 2000s and the withdrawal of rofecoxib [42–44]. However, the rate of increasing GI prophylaxis co-prescribing has slowed and reversed in 2017. Initial increases in bone prophylaxis reversed from 2008, with declining bisphosphonate co-prescribing while vitamin D-calcium co-prescribing plateaued around 50%. A similar trend in bisphosphonates was reported in Canada, USA and Australia following safety concerns [45–47]. With growing RA prevalence among the elderly who are most susceptible to multi-morbidity [48], renewed efforts to increase bone and GI prophylaxis are crucial, and must target extant as well as incident RA cases.

Study strengths included long-term follow-up of a large population-based cohort. Sensitivity analyses improved the specificity of the RA case definition and confirmed the robustness of the primary study findings. Sub-analyses assessing ≥180 days prescribing in a year enabled conservative estimates that confirmed findings. The matched non-RA cohort facilitated in discerning RA-specific prescribing patterns.

Study limitations include those common to EHR-based studies [49, 50]. RA definitions were affected by coding practices, including payment introduced for GPs in the UK in 2013, for diagnostic coding of RA patients [28], which correlates with a peak in incidence in this study, which we attempted to address with sensitivity analyses. It may have triggered incident coding of prevalent cases, where records had undifferentiated arthritis coded or free-text RA diagnostic references. The higher prevalence estimates post-2013 may therefore be more accurate. While data was utilized from a representative sample of UK GP practices, prescribing may differ between settings and countries. Importantly CPRD does not capture secondary care prescribing of conventional or biologic-DMARDs or intravenous bisphosphonates and denusomab so DMARDs and bone protectants were underestimated in this study. DMARD prescribing may have continued to rise through biologic availability in secondary care; however, these are typically second-line therapeutics and GP DMARD prescribing did not change across the life course, suggesting that the apparent plateau from 2009 requires investigation. Intramuscular, intravenous and intra-articular corticosteroids (which may also be given in secondary care) were not assessed, so the corticosteroid burden is also higher than we report and their use may have changed over time. Further, we did not examine all analgesics that may be used in RA management. Unascertainable prescription durations were set at 90 days, which may overestimate use given that the mode duration was 28 days. However, this affected <3% prescriptions, findings were consistent in sub-analyses of ≥180 days prescribing, and this should not affect interpretation of change over time. These long-term prescribing definitions should also allow for unused prescriptions, given the mode prescription duration. We did not examine change in corticosteroid dosages, which would inform understanding of exposure and medication tapering; however, toxicity is increased for all doses [8–11] and we showed prescribing for 15 years post-diagnosis, beyond the recommended duration for tapering. We could not distinguish where DMARDs were unsuitable or ineffective and long-term corticosteroids or NSAIDs formed part of an informed therapeutic approach, but such cases are uncommon [51] and DMARD prescribing was more common among RA patients with long-term corticosteroids.

Despite modern treatment strategies and increased DMARD prescription, long-term corticosteroid prescribing in RA patients remains substantial, especially among elderly patients, and persists once initiated. Long-term corticosteroid prescribing has clear implications for RA comorbidities and susceptibility to infection (of particular relevance during the COVID-19 pandemic). Rheumatologists need to understand the causes of persistent prescribing and develop alternative strategies of pain management.

## Acknowledgements

This work uses data provided by patients and collected by the NHS as part of their care and support. S.S.R.C. was supported by grant MR/L01629X from the Medical Research Council (MRC) Leeds Medical Bioinformatics Centre. P.G.C. and S.R.K. were in part supported by the National Institute for Health Research (NIHR) Leeds Biomedical Research Centre. The views expressed are those of the authors and not necessarily those of the NHS, the NIHR or the Department of Health. M.P.R. is currently employed by IQVIA, a research contract organization. The funders had no role in study design, data collection and analysis, decision to publish, or preparation of the manuscript. We thank Adam Keeley and the Data Analytics Team at the University of Leeds Institute for Data Analytics for their role in data collection and data management. S.S.R.C., M.H.B., S.R.K., M.P.R. and P.G.C. conceived the study. S.S.R.C., P.B. and M.P.R. designed the analyses and S.S.R.C. collected the data. S.S.R.C. conducted the analyses. S.S.R.C. generated the figures and tables and wrote the first draft of the manuscript. All authors critically revised the manuscript for intellectual content. S.S.R.C. had access to the data and acts as guarantor for this paper.

*Funding:* No specific funding was received from any funding agency in the public, commercial or not-for-profit sectors to carry out the work described in this manuscript.

**Transparency:** S.S.R.C. affirms that the manuscript is an honest, accurate and transparent account of the study being reported; no important aspects of the study have been omitted and any discrepancies from the study as originally planned have been explained.

*Disclosure statement:* All authors have completed the ICMJE uniform disclosure form at www.icmje.org/coi_disclosure.pdf and declare: S.S.R.C. reports a studentship grant from the Medical Research Council Leeds Medical Bioinformatics Centre, and S.R.K. and P.G.C. report grant support from the National Institute for Health Research Leeds Biomedical Research Centre. P.G.C. also reports personal fees from AbbVie, BMS, Gilead, GSK, Janssen, Novartis and Pfizer. All authors declare no further financial support; no financial relationships with any organizations that might have an interest in the submitted work in the previous 3 years; no other relationships or activities that could appear to have influenced the submitted work.

## Data availability statement 

The CPRD data were provided under a licence that does not permit sharing. The code lists used in definitions and the derived results are published in the manuscript and supplementary data.

## Supplementary data

Supplementary data are available at *Rheumatology* online.

## Supplementary Material

keaa892_Supplementary_Data
