# Supplementary material for: Changes in the pharmacological management of rheumatoid arthritis over two decades
Source: Rheumatology (Oxford). 2021 Jan 6;60(9):4141–51. doi: 10.1093/rheumatology/keaa892 (PMC8409998; doi:10.1093/rheumatology/keaa892)
Supplement: keaa892_Supplementary_Data [file keaa892_supplementary_data.docx]

# Supplementary Material

Table of Contents

[Glossary of Terms 2](#_Toc54618315)

[Supplementary Methods 3](#_Toc54618316)

[Supplementary Table S1. STROBE Checklist for cohort studies with reference to this study 3](#_Toc54618317)

[Supplementary Table S2. Read Codes used to determine diagnosis of rheumatoid arthritis 5](#_Toc54618318)

[Supplementary Table S3. Read Codes used to determine osteoporosis 6](#_Toc54618319)

[Supplementary Table S4. Drugs considered to determine prescribed medication 7](#_Toc54618320)

[Supplementary Data S1. Estimation of prescription duration 8](#_Toc54618321)

[Supplementary Data S2. Poisson regression modelling 8](#_Toc54618322)

[Supplementary Results 10](#_Toc54618323)

[Supplementary Figure S1. Study flow diagram of cohort selection (reported following STROBE guidelines (7)) 10](#_Toc54618324)

[Supplementary Table S5. Cohort baseline characteristics (at start of follow-up) 10](#_Toc54618325)

[Supplementary Table S6. Prevalence of RA by calendar year and sociodemographic factors (N = 7,532,147) 11](#_Toc54618326)

[Supplementary Table S7. Percentage of incident RA patients with ≥90 days annual prescribing in the 1-15 years post-diagnosis (N = 30,807) 12](#_Toc54618327)

[Supplementary Figure S2. Annual incidence rate of RA in females and males, 1997-2017 (N = 8,022,645) 12](#_Toc54618328)

[Supplementary Figure S3. Annual incidence rate of RA by age-group, 18-19 then 10 year increments, 1998-2017 (N = 8,021,209) 13](#_Toc54618329)

[Supplementary Figure S4. Annual incidence rate of RA by geographic region, 1998-2017 (N = 8,014,524) 13](#_Toc54618330)

[Supplementary Figure S5. Annual percentage prevalence of RA per age-group among patients aged 18-99, 1997-2017 (N = 7,531,867) 14](#_Toc54618331)

[Supplementary Figure S6. Annual percentage prevalence of RA among women and men, 1997-2017 (main analysis and sensitivity analyses 1 and 2) (N = 7,532,147) 14](#_Toc54618332)

[Supplementary Figure S7. Annual percentage prevalence of RA per geographic region, 1997-2017 (N = 7,521,506) 15](#_Toc54618333)

[Supplementary Figure S8. Percentage of RA patients with prescribed medication (≥1 prescriptions) per calendar year, 1998-2017 (N = 71,411) 15](#_Toc54618334)

[Supplementary Figure S9. Percentage of RA patients prescribed medication for ≥180 days per calendar year, 1998-2017 (N = 66,147) 16](#_Toc54618335)

[Supplementary Figure S10. Percentage of incident RA patients prescribed medication in the year post-diagnosis (≥1 prescriptions) per calendar year, 1998-2016 (N = 30,742) 16](#_Toc54618336)

[Supplementary Figure S11. Percentage of incident RA patients prescribed medication for ≥180 days in the year post-diagnosis, per calendar year, 1998-2016 (N = 29,164) 17](#_Toc54618337)

[Supplementary Figure S12. Percentage of RA patients with 1-12 annual oral prednisolone prescriptions per calendar year, 1998-2017 (N = 30,948) 17](#_Toc54618338)

[Supplementary Figure S14. Percentage of non-RA patients prescribed with medication for ≥90 days per calendar year, 1998-2017 (N = 195,636) 18](#_Toc54618339)

[Supplementary Figure S16. Percentage of RA patients prescribed with medication (≥1 prescription in year) in the 1-15 years following diagnosis (N = 31,768) 19](#_Toc54618340)

[Supplementary Figure S17. The percentage of RA patients with medication for ≥180 days in the 1-15 years following diagnosis (N = 29,790) 20](#_Toc54618341)

[Supplementary Figure S18. Percentage of female RA patients prescribed for ≥90 days with oral prednisolone and bisphosphonate per calendar year, by age-group (N = 14,314) 20](#_Toc54618342)

[Supplementary Figure S19. Percentage of female RA patients prescribed for ≥90 days with high dose (≥7.5 mg) oral prednisolone and bone protectant medication, by type of bone protectant medication and calendar year (N = 8,986) 21](#_Toc54618343)

[Supplementary Figure S20. Percentage of female RA patients prescribed for ≥90 days with low dose (<7.5 mg) oral prednisolone and bone protectant medication, by type of bone protectant medication and calendar year (N = 11,832) 21](#_Toc54618344)

[References 22](#_Toc54618345)

## Glossary of Terms

| CPRD | The Clinical Research Practice Datalink |
| --- | --- |
| DMARD | Disease modifying anti-rheumatic drug |
| IMD | Indices of multiple deprivation |
| NSAID | Non-steroidal anti-inflammatory drug |
| RA | Rheumatoid arthritis |
| STROBE | Strengthening the Reporting of Observational Studies in Epidemiology |

## Supplementary Methods

Supplementary Table S1. STROBE Checklist for cohort studies with reference to this study

|  | Item No | Recommendation | Page No |
| --- | --- | --- | --- |
| **Title and abstract** | 1 | (*a*) Indicate the study’s design with a commonly used term in the title or the abstract | 3 |
|  |  | (*b*) Provide in the abstract an informative and balanced summary of what was done and what was found | 3 |
| Introduction | | |  |
| Background/rationale | 2 | Explain the scientific background and rationale for the investigation being reported | 6 |
| Objectives | 3 | State specific objectives, including any prespecified hypotheses | 6 |
| Methods | | |  |
| Study design | 4 | Present key elements of study design early in the paper | 7 |
| Setting | 5 | Describe the setting, locations, and relevant dates, including periods of recruitment, exposure, follow-up, and data collection | 7 |
| Participants | 6 | (*a*) Give the eligibility criteria, and the sources and methods of selection of participants. Describe methods of follow-up | 7 |
|  |  | (*b*) For matched studies, give matching criteria and number of exposed and unexposed | 7 |
| Variables | 7 | Clearly define all outcomes, exposures, predictors, potential confounders, and effect modifiers. Give diagnostic criteria, if applicable | 8 |
| Data sources/ measurement | 8* | For each variable of interest, give sources of data and details of methods of assessment (measurement). Describe comparability of assessment methods if there is more than one group | 7, 8 |
| Bias | 9 | Describe any efforts to address potential sources of bias | 8 |
| Study size | 10 | Explain how the study size was arrived at | 7 |
| Quantitative variables | 11 | Explain how quantitative variables were handled in the analyses. If applicable, describe which groupings were chosen and why | 8 |
| Statistical methods | 12 | (*a*) Describe all statistical methods, including those used to control for confounding | 8-9 |
|  |  | (*b*) Describe any methods used to examine subgroups and interactions | 8-9 |
|  |  | (*c*) Explain how missing data were addressed | 9 |
|  |  | (*d*) If applicable, explain how loss to follow-up was addressed | NA |
|  |  | (*e*) Describe any sensitivity analyses | 9 |
| Results | | |  |
| Participants | 13* | (a) Report numbers of individuals at each stage of study—eg numbers potentially eligible, examined for eligibility, confirmed eligible, included in the study, completing follow-up, and analysed | 10, 15, Sup. Fig. 1, |
|  |  | (b) Give reasons for non-participation at each stage | Sup. Fig. 1 |
|  |  | (c) Consider use of a flow diagram | 7, Sup. Fig. 1 |
| Descriptive data | 14* | (a) Give characteristics of study participants (eg demographic, clinical, social) and information on exposures and potential confounders | 10, Sup. Table 5 |
|  |  | (b) Indicate number of participants with missing data for each variable of interest | 10 |
|  |  | (c) Summarise follow-up time (eg, average and total amount) | 15 |
| Outcome data | 15* | Report numbers of outcome events or summary measures over time | 10-15 |
| Main results | 16 | (*a*) Give unadjusted estimates and, if applicable, confounder-adjusted estimates and their precision (eg, 95% confidence interval). Make clear which confounders were adjusted for and why they were included | 11-14 |
|  |  | (*b*) Report category boundaries when continuous variables were categorized | 7 |
|  |  | (*c*) If relevant, consider translating estimates of relative risk into absolute risk for a meaningful time period | NA |
| Other analyses | 17 | Report other analyses done—eg analyses of subgroups and interactions, and sensitivity analyses | 10-14 |
| Discussion | | |  |
| Key results | 18 | Summarise key results with reference to study objectives | 16 |
| Limitations | 19 | Discuss limitations of the study, taking into account sources of potential bias or imprecision. Discuss both direction and magnitude of any potential bias | 18 |
| Interpretation | 20 | Give a cautious overall interpretation of results considering objectives, limitations, multiplicity of analyses, results from similar studies, and other relevant evidence | 17, 18 |
| Generalisability | 21 | Discuss the generalisability (external validity) of the study results | 16, 17 |
| Other information | | |  |
| Funding | 22 | Give the source of funding and the role of the funders for the present study and, if applicable, for the original study on which the present article is based | 19 |

Supplementary Table S2. Read Codes used to determine diagnosis of rheumatoid arthritis

| **Code** | **Term Description** |
| --- | --- |
| 14G1. | H/O: rheumatoid arthritis |
| 38DZ. | Disease activity score in rheumatoid arthritis |
| 38DZ0 | Disease activity score 28 joint in rheumatoid arthritis |
| 38Vs. | Rheumatoid Arthritis Impact of Disease questionnaire |
| 66HB0 | Rheumatoid arthritis annual review |
| 7P203 | Delivery of rehabilitation for rheumatoid arthritis |
| 9hR.. | Exception reporting: rheumatoid arthritis quality indicators |
| 9hR0. | Excepted from rheumatoid arthritis quality indicators: patient unsuitable |
| 9hR1. | Excepted from rheumatoid arthritis quality indicators: informed dissent |
| 9mM.. | Rheumatoid arthritis monitoring invitation |
| 9mM0. | Rheumatoid arthritis monitoring invitation first letter |
| 9mM1. | Rheumatoid arthritis monitoring invitation second letter |
| 9mM2. | Rheumatoid arthritis monitoring invitation third letter |
| 9mM3. | Rheumatoid arthritis monitoring verbal invitation |
| 9mM4. | Rheumatoid arthritis monitoring telephone invitation |
| F3712 | Polyneuropathy in rheumatoid arthritis |
| F3964 | Myopathy due to rheumatoid arthritis |
| N040. | Rheumatoid arthritis |
| N0400 | Rheumatoid arthritis of cervical spine |
| N0401 | Other rheumatoid arthritis of spine |
| N0402 | Rheumatoid arthritis of shoulder |
| N0403 | Rheumatoid arthritis of sternoclavicular joint |
| N0404 | Rheumatoid arthritis of acromioclavicular joint |
| N0405 | Rheumatoid arthritis of elbow |
| N0406 | Rheumatoid arthritis of distal radio-ulnar joint |
| N0407 | Rheumatoid arthritis of wrist |
| N0408 | Rheumatoid arthritis of metacarpophalangeal joint |
| N0409 | Rheumatoid arthritis of proximal interphalangeal joint of finger |
| N040A | Rheumatoid arthritis of distal interphalangeal joint of finger |
| N040B | Rheumatoid arthritis of hip |
| N040C | Rheumatoid arthritis of sacro-iliac joint |
| N040D | Rheumatoid arthritis of knee |
| N040E | Rheumatoid arthritis of tibio-fibular joint |
| N040F | Rheumatoid arthritis of ankle |
| N040G | Rheumatoid arthritis of subtalar joint |
| N040H | Rheumatoid arthritis of talonavicular joint |
| N040J | Rheumatoid arthritis of other tarsal joint |
| N040K | Rheumatoid arthritis of 1st metatarsophalangeal joint |
| N040L | Rheumatoid arthritis of lesser metatarsophalangeal joint |
| N040M | Rheumatoid arthritis of interphalangeal joint of toe |
| N040P | Seronegative rheumatoid arthritis |
| N040S | Rheumatoid arthritis - multiple joint |
| N040T | Flare of rheumatoid arthritis |
| N047. | Seropositive errosive rheumatoid arthritis |
| N04X. | Seropositive rheumatoid arthritis, unspecified |
| Nyu10 | [X]Rheumatoid arthritis with involvement of other organs or systems |
| Nyu11 | [X]Other seropositive rheumatoid arthritis |
| Nyu12 | [X]Other specified rheumatoid arthritis |
| Nyu1G | [X]Seropositive rheumatoid arthritis, unspecified |

Supplementary Table S3. Read Codes used to determine osteoporosis

| **Code** | **Term Description** |
| --- | --- |
| N331L00 | collapse of vertebra due to osteoporosis nos |
| N331J00 | collapse of lumbar vertebra due to osteoporosis |
| N331M00 | fragility fracture due to unspecified osteoporosis |
| N331900 | osteoporosis + pathological fracture thoracic vertebrae |
| N331800 | osteoporosis + pathological fracture lumbar vertebrae |
| NyuB800 | [x]unspecified osteoporosis with pathological fracture |
| N331K00 | collapse of thoracic vertebra due to osteoporosis |
| N331600 | idiopathic osteoporosis with pathological fracture |
| N331300 | osteoporosis of disuse with pathological fracture |
| N331.14 | osteoporotic vertebral collapse |
| N331H00 | collapse of cervical vertebra due to osteoporosis |
| N331A00 | osteoporosis + pathological fracture cervical vertebrae |
| N331M11 | minimal trauma fracture due to unspecified osteoporosis |
| 66aB.00 | osteoporosis - no treatment response |
| 66aA.00 | osteoporosis - treatment response |
| N330.00 | Osteoporosis |
| N330B00 | vertebral osteoporosis |
| N330000 | osteoporosis, unspecified |
| N330C00 | osteoporosis localized to spine |
| 66a9.00 | osteoporosis - falls prevention |
| N330z00 | osteoporosis nos |
| N374600 | osteoporotic kyphosis |
| N330300 | idiopathic osteoporosis |
| N330800 | localized osteoporosis – lequesne |
| N330400 | dissuse osteoporosis |
| 9hP..00 | exception reporting: osteoporosis quality indicators |
| N330100 | senile osteoporosis |
| N331B00 | postmenopausal osteoporosis with pathological fracture |
| N330200 | postmenopausal osteoporosis |
| N331500 | drug-induced osteoporosis with pathological fracture |
| N330500 | drug-induced osteoporosis |
| N330D00 | osteoporosis due to corticosteroids |
| N330A00 | osteoporosis in endocrine disorders |
| N331200 | postoophorectomy osteoporosis with pathological fracture |
| N330600 | postoophorectomy osteoporosis |
| N331400 | postsurgical malabsorption osteoporosis with path fracture |
| N330700 | postsurgical malabsorption osteoporosis |
| NyuB000 | [x]other osteoporosis with pathological fracture |
| NyuB100 | [x]other osteoporosis |
| N330900 | osteoporosis in multiple myelomatosis |
| NyuB200 | [x]osteoporosis in other disorders classified elsewhere |

Supplementary Table S4. Drugs considered to determine prescribed medication

| **Medication** |
| --- |
| **Disease-modifying anti-rheumatic drugs**  Route: gastroenteral, intraarterial, intravenous, oral, subcutaneous  Term: Abatacept; Adalimumab; Azathioprine; Baricitinib; Certolizumab; Ciclosporin / cyclosporine; Cyclophosphamid/e; Etanercept; Gold injections / injectable gold / sodium aurothiomalate; Golimumab; Hydroxychloroquine; Infliximab; Leflunomide; Methotrexate; Mycophenolate / mycophenalte mofetil; Penicillamine; Rituximab; Sarilumab; Sulfasalazine; Tocilizumab; Tofacitinib; Ustekinumab |
| **Oral corticosteroids**  Route: oral  Term: Betamethasolone; Betamethasone; Bethamethasone; Budesonide; lobetasone; Cortisone; Deflazacort; Dexamethasone; Fluorometholone; Hydrocortisone; Loteprednol; Methylprednisolone; Prednisolone; Prednisone; Rimexolone; Triamcinolone |
| **Non-steroidal anti-inflammatory drugs**  Route: cutaneous, oral, rectal, topical, transdermal  Term: Aceclofenac; Acemetacin; Celecoxib; Dexibuprofen; Dexketoprofen Trometamol; Diclofenac potassium; Diclofenac sodium; Misoprostol; Diflunisal; Etodolac; Etoricoxib; Fenbufen; Fenoprofen calcium; Flurbiprofen; Ibuprofen; Indometacin; Ketoprofen; Lornoxicam; Magnesium trisilicate; Mefenamic acid; Meloxicam; Nabumetone; Naproxen; Naproxen sodium; Phenylbutazone; Piroxicam; Piroxicam betadex; Salsalate; Sulindac; Tenoxicam; Tiaprofenic acid; Tolmetin sodium |
| **Prednisolone**  Route: oral  Term: Methylprednisolone; Prednisolone; Prednisone |
| **Proton pump inhibitors**  Route: oral  Term: Esomeprazole; Lansoprazole; Omeprazole; Pantoprazole; Rabeprazole |
| **Bisphosphonates**  Route: oral  Term: Alendronate sodium; Alendronic acid; Ibandronic sodium monohydrate; Risedronate sodium |
| **Vitamin D**  Route: oral  Term: Alfacalcidol; Calcitriol; Colecalciferol; Dihydrotechysterol; Ergocalciferol; Paricalcitrol |
| **Calcium**  Route: oral  Term: Calcium; Calcium carbonate; Calcium chloride dihydrate; Calcium gluconate; Calcium lactate; Tricalcium phosphate; and excluding the phrase ‘indigestion’  BNF chapter: contains the term vitamin, supplement or not stated |

Note: Prescriptions are defined in CPRD using British National Formulary (BNF) terms and were selected by consideration of BNF chapters, Yorkshire DMARD guidelines and drug lists on the NHS and Versus Arthritis websites (1-4).

### Supplementary Data S1. Estimation of prescription duration

Prescription duration was calculated using an algorithm reported by Partington et al. (2018) (5):

1. “If available, duration of each prescription recorded in CPRD was used.
2. If not, the duration of each prescription was the lowest of,
   1. the quantity of medication prescribed; or
   2. the gap until the next prescription [*of that drug group*] (if this was <90days); or
   3. the quantity of medication prescribed divided by the daily dose (if this was recorded).
3. If the duration was still missing, it was replaced with,
   1. the average of that patient’s duration for other prescriptions of the same drug with the same strength (if present); or
   2. the average duration for all other patients’ prescriptions of the same drug with the same strength.
4. If prescription duration was >90 days, it was replaced as 90 days.”

The 90 day period is also used in other CPRD-based medication-use studies (6) and was the longest of the commonly prescribed medication durations in this study.

Where multiple prescriptions existed on one day, one contributed to the prescription duration calculation per medication. This avoided over-counting from misplaced prescriptions yet is conservative where patients retain prescriptions to issue as required.

### Supplementary Data S2. Poisson regression modelling

The following analysis was run using R version 3.6.1, for all RA patients and for incident RA patients in the year following diagnosis, and the subset of each with IMD recorded.

Fixed effects Poisson regression

(chosen for: long-term corticosteroid prescribing in the year following incident diagnosis in the full cohort (Table 2); long-term corticosteroid prescribing for all RA patients in the IMD subset; long-term corticosteroid prescribing in the year following incident diagnosis in the IMD subset; long-term NSAID prescribing in the year following incident diagnosis in the IMD subset)

summary(model1 <- glm(longTermPrescribing ~ Year + Sex + AgeGroup + imd2015_5 + offset(logPopulation), data = allData, family = poisson(link = "log")))

(chosen for: long-term NSAID prescribing for all RA patients in the IMD subset)

summary(model2 <- glm(longTermPrescribing ~ Year + Sex + AgeGroup + imd2015_5 + offset(logPopulation), data = subsetIMD, family = poisson(link = "log")))

Fixed effects quasi-Poisson regression

(chosen for long-term corticosteroid and NSAID prescribing in all RA patients (Table 2))

summary(model3 <- glm(longTermPrescribing ~ Year + Sex + AgeGroup + imd2015_5 + offset(logPopulation), data = allData, family = quasipoisson(link = "log")))

summary(model4 <- glm(longTermPrescribing ~ Year + Sex + AgeGroup + imd2015_5 + offset(logPopulation), data = subsetIMD, family = quasipoisson(link = "log")))

Random effects Poisson regression

(chosen for long-term NSAID prescribing in the year following incident diagnosis (Table 2))

summary(model5 <- glmer(longTermPrescribing ~ Year + Sex + AgeGroup + offset(logpopn) + (1 | ProjectPracID), data = allData, family = poisson(link = "log"), nAGQ=0 ))

summary(model6 <- glmer(longTermPrescribing ~ Year + Sex + AgeGroup + + imd2015 + offset(logpopn) + (1 | ProjectPracID), data = subsetIMD, family = poisson(link = "log"), nAGQ=0 ))

Zero-inflation Poisson regression

summary(model7 <- mixed_model(longTermPrescribing ~ Year + Sex + AgeGroup + offset(logpopn), random= ~1 | ProjectPracID, data = allData, family = zi.poisson(), zi_fixed=~Year, zi_random= ~1 | ProjectPracID))

summary(model8 <- mixed_model(longTermPrescribing ~ Year + Sex + AgeGroup + imd2015 + offset(logpopn), random= ~1 | ProjectPracID, data = allData, family = zi.poisson(), zi_fixed=~Year, zi_random= ~1 | ProjectPracID))

For each model, robust standard errors and P values with 95% confidence intervals were obtained using the parameter estimates and their standard errors (presented for model1 as an example)

cov.m1<-vcovHC(model1, type="HC0")

std.err<-sqrt(diag(cov.m1))

r.est<-cbind(Estimate=coef(model1), "Robust SE" = std.err, "P(>|z|)" = 2*pnorm(abs(coef(model1)/std.err), lower.tail=FALSE), LL=coef(model1)-1.96*std.err, UL = coef(model1) + 1.96*std.err)

r.est

Incidence rate ratios were calculated, with the Delta method to define standard error (presented for model1 as an example)

g<-deltamethod(list(~exp(x1),~exp(x2),~exp(x3),~exp(x4)), coef(model1), cov.model1)

rexp.est<-exp(r.est[,-3])

rexp.est[, "Robust SE"] <- g

rexp.est

Fixed and random effects models were compared using the Hausman test:

library(plm)

phtest(model1, model5)

## Supplementary Results


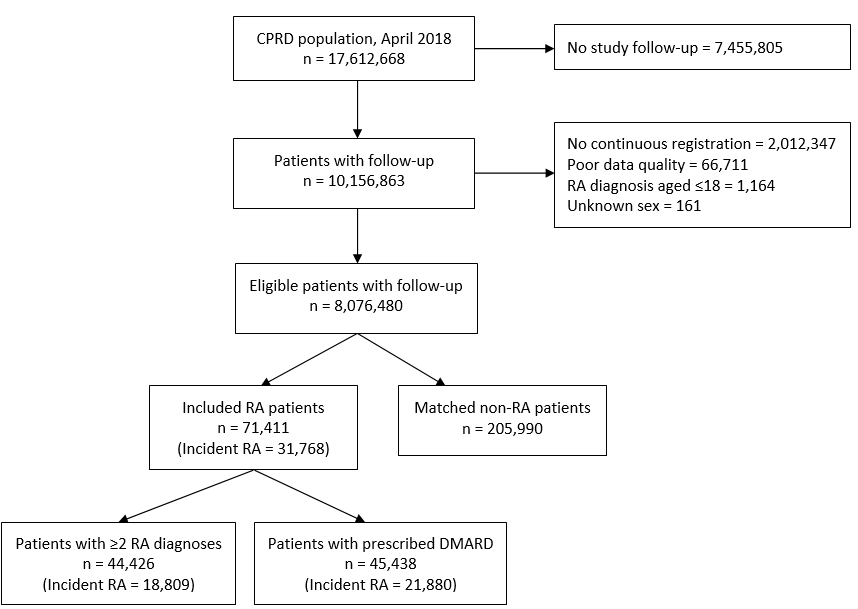
Supplementary Figure S1. Study flow diagram of cohort selection (reported following STROBE guidelines (7))

Supplementary Table S5. Cohort baseline characteristics (at start of follow-up)

|  | **All RA patients**  **(N = 71,411)** | **Incident RA patients**  **(N = 31,768)** | **Non-RA patients**  **(N = 205,990)** |
| --- | --- | --- | --- |
| **Median age (years) [IQR]** | 57 [23] | 61 [22] | 60 [22] |
| **Females (%)** | 49,974 (70.0) | 21,464 (67.6) | 140,685 (68.3) |
| **Median follow-up duration (years) [IQR]** | 5.1 [7.6] | 4.4 [6.1] | 3.6 [5.4] |
| **Asthma (%)** | 10,083 (14.1) | 5,130 (16.1) | 23,672 (11.5) |
| **COPD (%)** | 4,302 (6.0) | 2,101 (6.6) | 7,431 (3.6) |
| **Osteoarthritis (%)** | 18,551 (26.0) | 9,632 (30.3) | 31,121 (15.1) |

Supplementary Table S6. Prevalence of RA by calendar year and sociodemographic factors (N = 7,532,147)

|  | Percentage prevalence (95% CI) |
| --- | --- |
| Overall | 0.89 (0.88 to 0.89) |
| Year |  |
| 1998 | 0.70 (0.68 to 0.71) |
| 1999 | 0.73 (0.71 to 0.74) |
| 2000 | 0.73 (0.72 to 0.74) |
| 2001 | 0.73 (0.71 to 0.74) |
| 2002 | 0.74 (0.73 to 0.75) |
| 2003 | 0.75 (0.74 to 0.76) |
| 2004 | 0.76 (0.75 to 0.77) |
| 2005 | 0.78 (0.77 to 0.79) |
| 2006 | 0.78 (0.78 to 0.79) |
| 2007 | 0.79 (0.78 to 0.80) |
| 2008 | 0.79 (0.78 to 0.80) |
| 2009 | 0.79 (0.78 to 0.80) |
| 2010 | 0.79 (0.78 to 0.80) |
| 2011 | 0.79 (0.78 to 0.80) |
| 2012 | 0.79 (0.78 to 0.80) |
| 2013 | 0.79 (0.78 to 0.80) |
| 2014 | 0.86 (0.84 to 0.87) |
| 2015 | 0.88 (0.87 to 0.89) |
| 2016 | 0.93 (0.91 to 0.94) |
| 2017 | 0.91 (0.90 to 0.93) |
| Sex |  |
| Female | 1.22 (1.21 to 1.23) |
| Male | 0.54 (0.54 to 0.55) |
| Age group |  |
| 18-29 | 0.05 (0.05 to 0.05) |
| 30-39 | 0.20 (0.19 to 0.21) |
| 40-49 | 0.49 (0.48 to 0.50) |
| 50-59 | 1.00 (0.99 to 1.01) |
| 60-69 | 1.67 (1.65 to 1.69) |
| 70-79 | 2.26 (2.23 to 2.28) |
| 80-89 | 2.33 (2.29 to 2.36) |
| 90-99 | 1.72 (1.67 to 1.78) |
| Geographical area |  |
| North East | 0.93 (0.87 to 0.98) |
| North West | 0.94 (0.92 to 0.95) |
| Yorkshire & The Humber | 1.04 (1.00 to 1.07) |
| East Midlands | 0.90 (0.86 to 0.93) |
| West Midlands | 0.92 (0.89 to 0.94) |
| East of England | 0.92 (0.90 to 0.95) |
| South West | 0.94 (0.92 to 0.96) |
| South Central | 0.78 (0.76 to 0.80) |
| London | 0.66 (0.65 to 0.68) |
| South East Coast | 0.88 (0.86 to 0.90) |
| Northern Ireland | 1.06 (1.01 to 1.10) |
| Scotland | 0.92 (0.90 to 0.94) |
| Wales | 1.00 (0.98 to 1.03) |

Supplementary Table S7. Percentage of incident RA patients with ≥90 days annual prescribing in the 1-15 years post-diagnosis (N = 30,807)

| Year post-diagnosis | Percentage with prescribing (95% CI) | | |
| --- | --- | --- | --- |
|  | DMARD | Corticosteroid | NSAID |
| 1 | 54.4 (53.9 to 55.0) | 22.2 (21.7 to 22.6) | 41.2 (40.6 to 41.7) |
| 2 | 55.1 (54.5 to 55.7) | 19.2 (18.8 to 19.7) | 36.0 (35.5 to 36.6) |
| 3 | 54.2 (53.6 to 54.8) | 17.9 (17.4 to 18.4) | 34.3 (33.7 to 34.9) |
| 4 | 53.5 (52.8 to 54.2) | 17.1 (16.6 to 17.6) | 33.4 (32.7 to 34.0) |
| 5 | 54.0 (53.2 to 54.7) | 17.3 (16.7 to 17.9) | 33.6 (32.8 to 34.3) |
| 6 | 53.8 (53.0 to 54.7) | 17.4 (16.8 to 18.1) | 33.4 (32.6 to 34.2) |
| 7 | 53.4 (52.4 to 54.3) | 17.9 (17.2 to 18.6) | 32.7 (31.9 to 33.6) |
| 8 | 52.7 (51.7 to 53.7) | 17.2 (16.5 to 18.0) | 32.5 (31.6 to 33.4) |
| 9 | 52.3 (51.2 to 53.4) | 16.8 (16.0 to 17.6) | 30.9 (29.9 to 31.9) |
| 10 | 52.8 (51.6 to 54.0) | 16.4 (15.5 to 17.3) | 31.0 (29.9 to 32.2) |
| 11 | 52.1 (50.7 to 53.5) | 16.3 (15.3 to 17.3) | 31.1 (29.8 to 32.3) |
| 12 | 51.7 (50.1 to 53.3) | 15.8 (14.6 to 17.0) | 29.8 (28.3 to 31.3) |
| 13 | 51.8 (50.0 to 53.7) | 15.3 (14.0 to 16.7) | 30.2 (28.5 to 31.9) |
| 14 | 52.6 (50.4 to 54.8) | 15.9 (14.3 to 17.5) | 28.8 (26.8 to 30.8) |
| 15 | 51.6 (48.9 to 54.3) | 16.9 (14.9 to 18.9) | 28.4 (25.9 to 30.8) |

Supplementary Figure S2. Annual incidence rate of RA in females and males, 1997-2017 (N = ****8,022,645)****


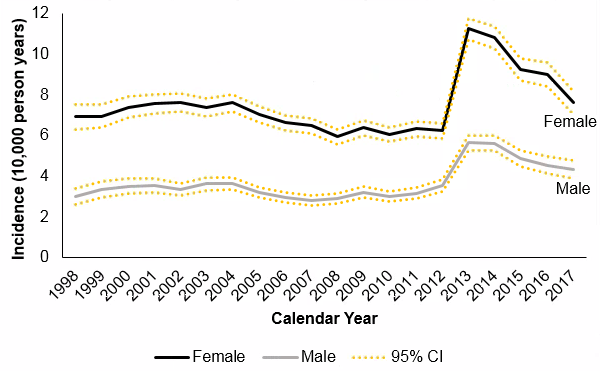


Supplementary Figure S3. Annual incidence rate of RA by age-group, 18-19 then 10 year increments, 1998-2017 (N = ****8,021,209)****


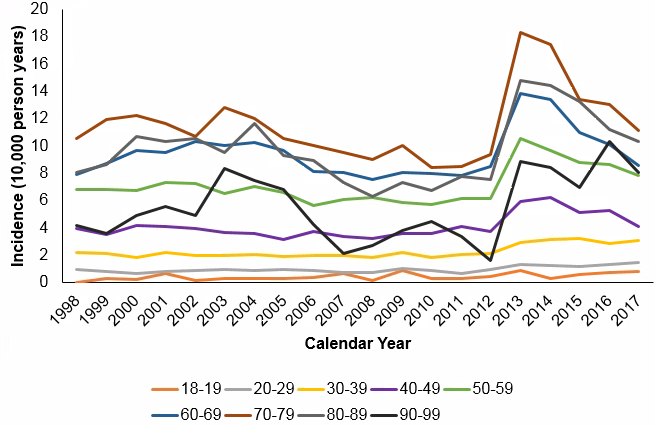


Supplementary Figure S4. Annual incidence rate of RA by geographic region, 1998-2017 (N = 8,014,524)


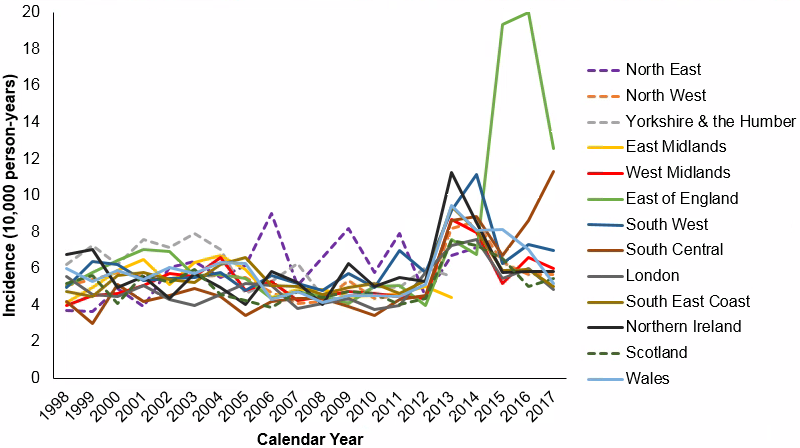


Supplementary Figure S5. Annual percentage prevalence of RA per age-group among patients aged 18-99, 1997-2017 (N = 7,531,867)


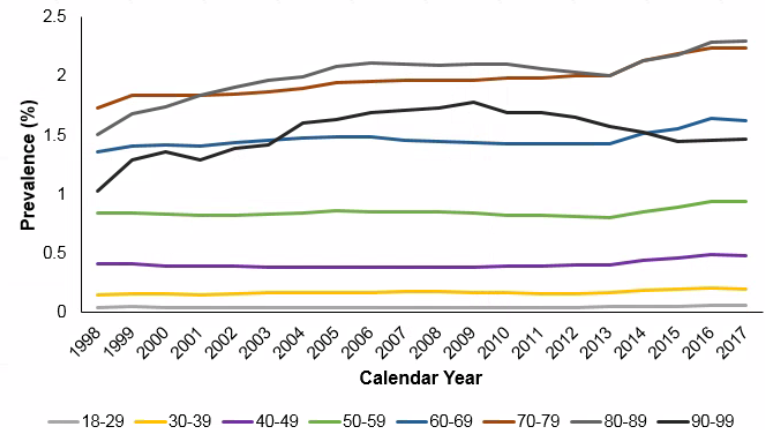


Supplementary Figure S6. Annual percentage prevalence of RA among women and men, 1997-2017 (main analysis and sensitivity analyses 1 and 2) (N = 7,532,147)


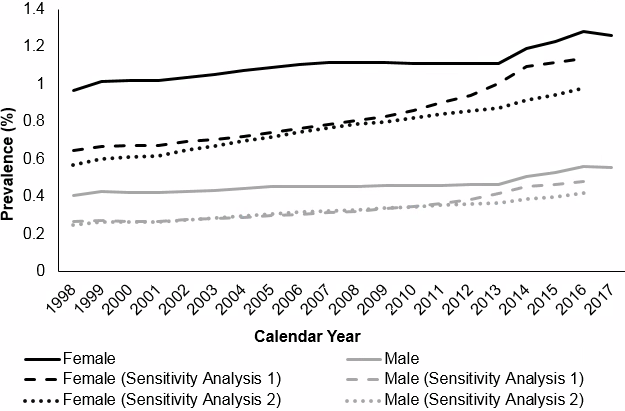


Supplementary Figure S7. Annual percentage prevalence of RA per geographic region, 1997-2017 (N = 7,521,506)


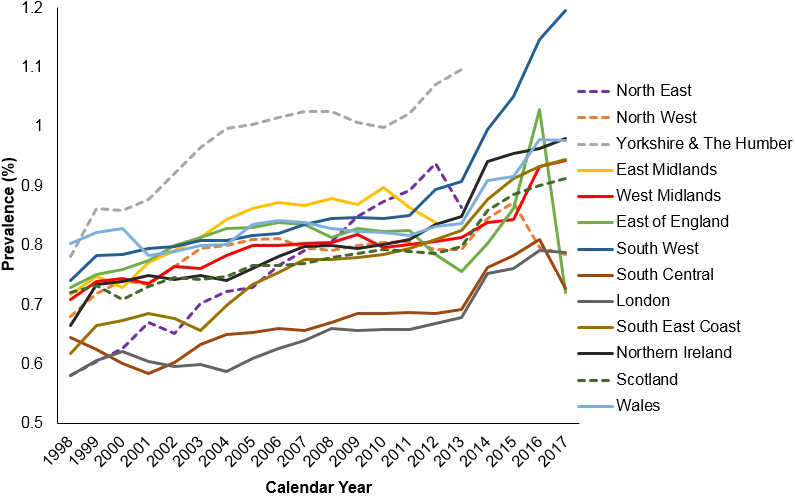


Supplementary Figure S8. Percentage of RA patients with prescribed medication (≥1 prescriptions) per calendar year, 1998-2017 (N = 71,411)


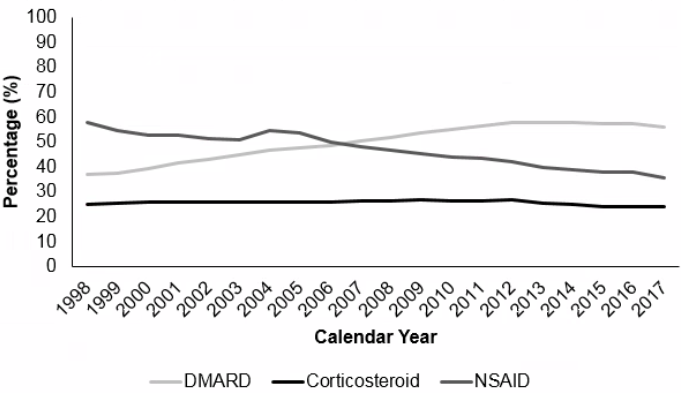


Supplementary Figure S9. Percentage of RA patients prescribed medication for ≥180 days per calendar year, 1998-2017 (N = 66,147)


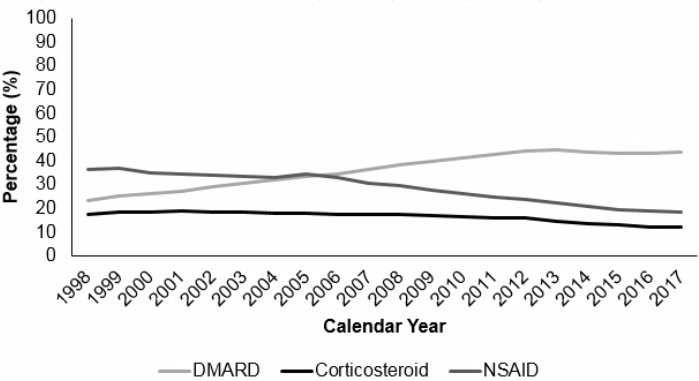


Supplementary Figure S10. Percentage of incident RA patients prescribed medication in the year post-diagnosis (≥1 prescriptions) per calendar year, 1998-2016 (N = 30,742)


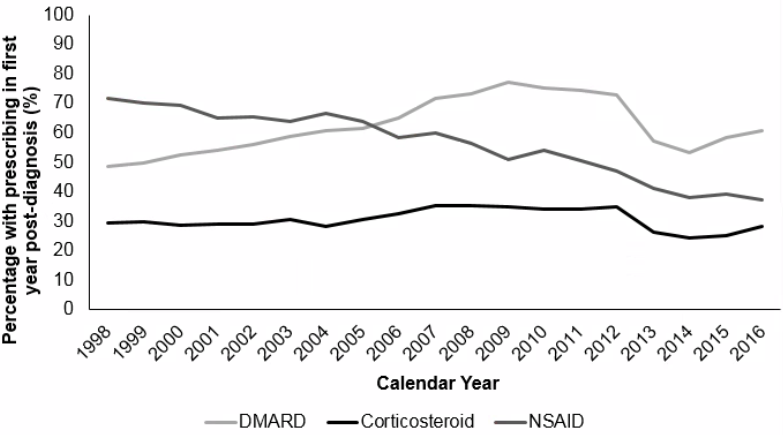


Supplementary Figure S11. Percentage of incident RA patients prescribed medication for ≥180 days in the year post-diagnosis, per calendar year, 1998-2016 (N = 29,164)


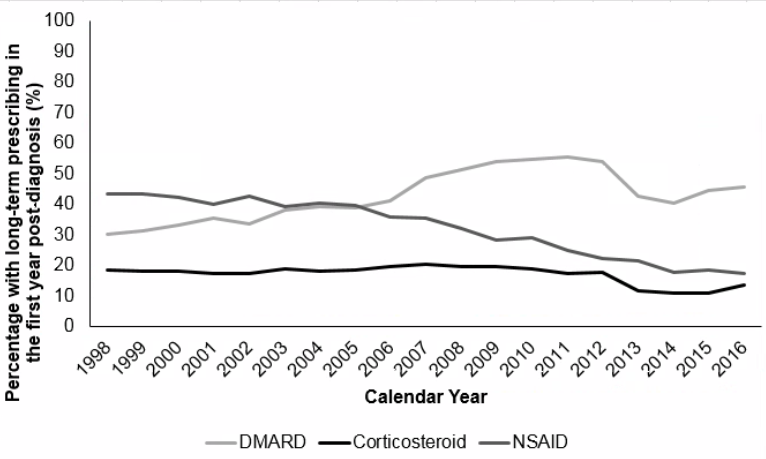


Supplementary Figure S12. Mean annual prescription count among all RA patients and among RA patients with ≥1 prescription in a given year, 1998-2017 (N = 71,411)


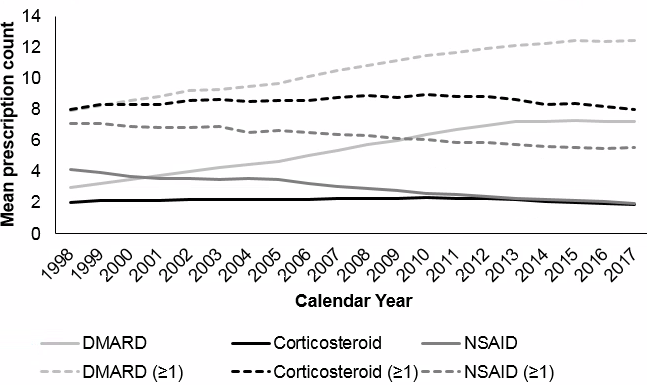


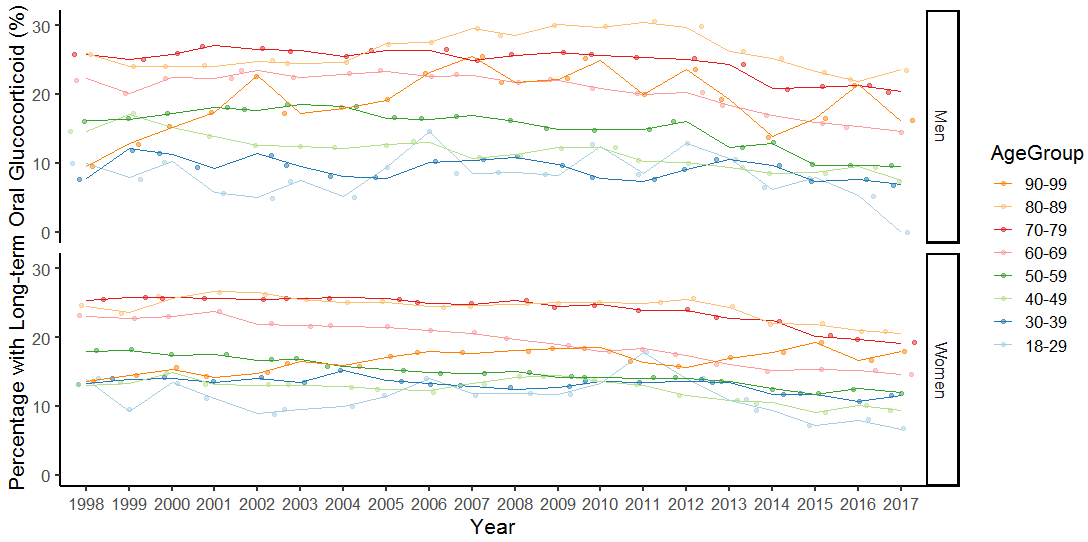


Supplementary Figure S13. Percentage of RA patients prescribed with oral corticosteroids for ≥90 days by age-group and sex, 1998-2017 (N = 21,726)

Supplementary Figure S14. Percentage of non-RA patients prescribed with medication for ≥90 days per calendar year, 1998-2017 (N = ****195,636)****


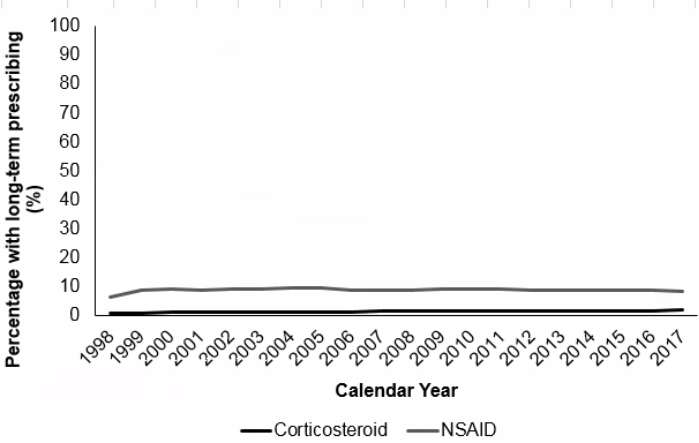


Supplementary Figure S15. The percentage of RA patients with medication for ≥90 days in the 1-15 years following diagnosis (N = 30,807)


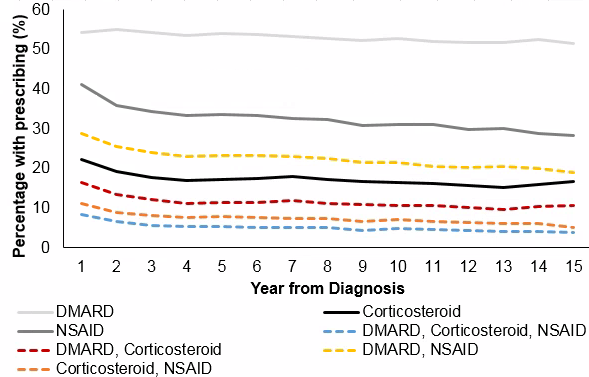


Supplementary Figure S16. Percentage of RA patients prescribed with medication (≥1 prescription in year) in the 1-15 years following diagnosis (N = 31,768)


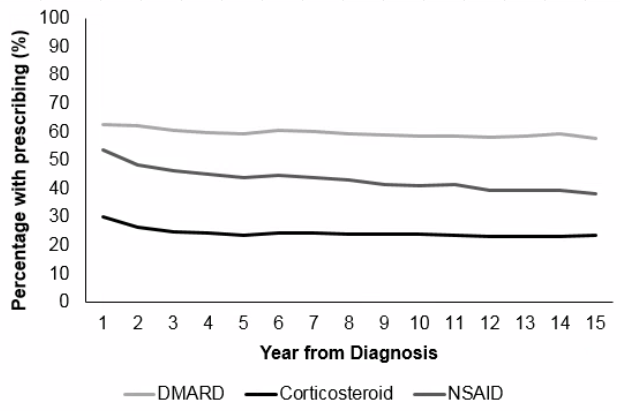


Supplementary Figure S17. The percentage of RA patients with medication for ≥180 days in the 1-15 years following diagnosis (N = 29,790)


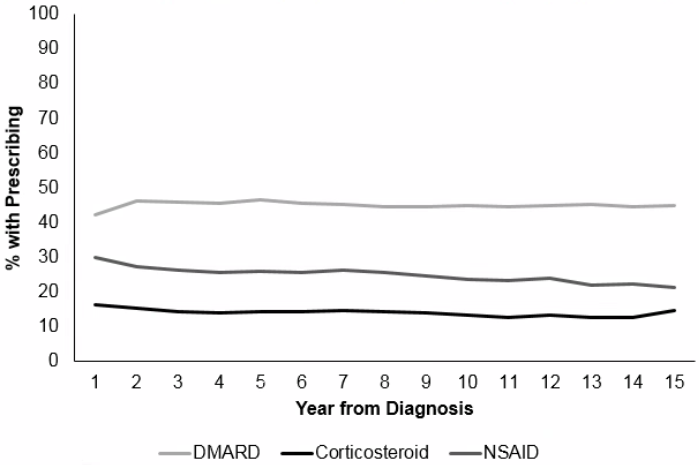


Supplementary Figure S18. Percentage of female RA patients prescribed for ≥90 days with oral prednisolone and bisphosphonate per calendar year, by age-group (N = 14,314)


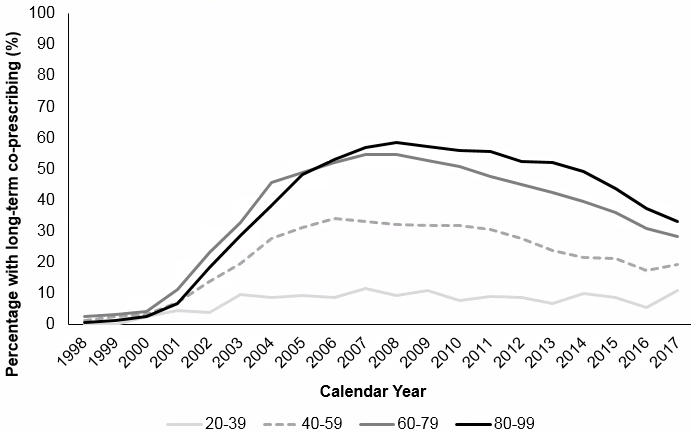


Supplementary Figure S19. Percentage of female RA patients prescribed for ≥90 days with high dose (≥7.5 mg) oral prednisolone and bone protectant medication, by type of bone protectant medication and calendar year (N = 5,952)


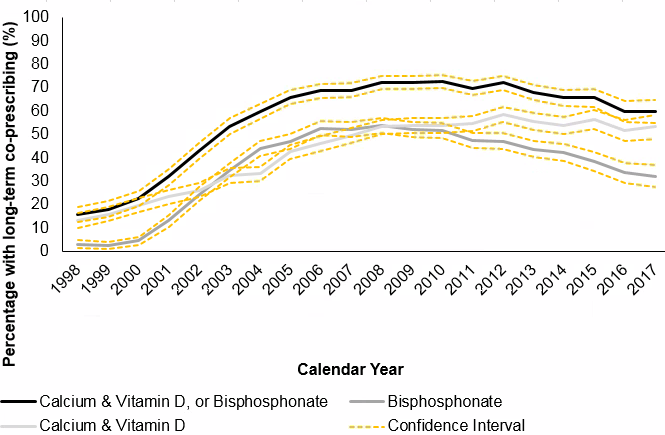


Supplementary Figure S20. Percentage of female RA patients prescribed for ≥90 days with low dose (<7.5 mg) oral prednisolone and bone protectant medication, by type of bone protectant medication and calendar year (N = 13,061)


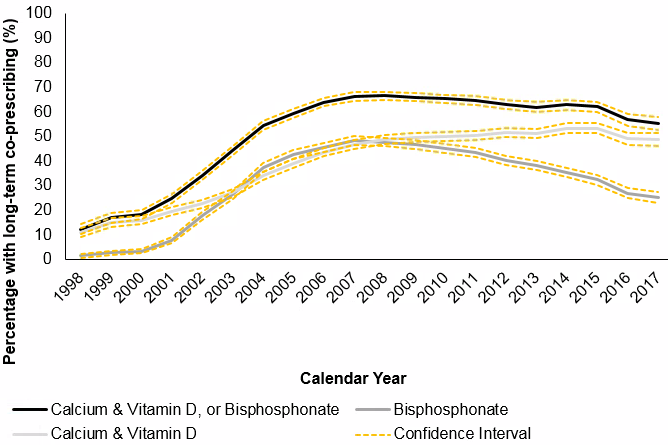


## References

1. Arthritis, V. [Online]. [Accessed 02.07.2019]. Available from: <https://www.versusarthritis.org/>

2. *Yorkshire rheumatology regional guidelines for the monitoring of adult patients on conventional disease modifying drugs, biologic drugs and targeted synthetic drugs: Version 7.* [Online]. 2019. [Accessed 02.07.2019]. Available from: <https://www.bradfordhospitals.nhs.uk/wp-content/uploads/2019/07/YORKSHIRE-DMARD-GUIDELINES-2019-FINAL.pdf>

3. Health, D.o. *NHS.* [Online]. [Accessed 02.07.2019]. Available from: [www.nhs.uk](https://leeds365-my.sharepoint.com/personal/medsacr_leeds_ac_uk/Documents/MSK%20PhD/RA/Paper/www.nhs.uk)

4. *British National Formulary.* [Online]. [Accessed 02.07.2019]. Available from: <https://www.medicinescomplete.com/#/>

5. Partington, R.J., Muller, S., Helliwell, T., Mallen, C.D. and Sultan, A.A. Incidence, prevalence and treatment burden of polymyalgia rheumatica in the UK over two decades: a population-based study. *Ann Rheum Dis.* 2018, **77**(12), pp.1750-1756.

6. Vinogradova, Y., Coupland, C., Brindle, P. and Hippisley-Cox, J. Discontinuation and restarting in patients on statin treatment: prospective open cohort study using a primary care database. *Bmj.* 2016, **353**, p.i3305.

7. Von Elm, E., Altman, D.G., Egger, M., Pocock, S.J., Gøtzsche, P.C. et al. The Strengthening the Reporting of Observational Studies in Epidemiology (STROBE) statement: guidelines for reporting observational studies. 2007, **147**(8), pp.573-577.
